# Bridging structural biology and clinical research through in-tissue cryo-electron tomography

**DOI:** 10.1038/s44318-024-00216-z

**Published:** 2024-09-16

**Authors:** Kathryn Kixmoeller, Benjamin C Creekmore, Edward B Lee, Yi-Wei Chang

**Affiliations:** 1 Department of Biochemistry and Biophysics, Perelman School of Medicine, University of Pennsylvania, Philadelphia, PA USA; 2 Institute of Structural Biology, Perelman School of Medicine, University of Pennsylvania, Philadelphia, PA USA; 3 Translational Neuropathology Research Laboratory, Department of Pathology and Laboratory Medicine, Perelman School of Medicine, University of Pennsylvania, Philadelphia, PA USA

**Keywords:** Structural Biology

## Abstract

This commentary of the *Sparks of Science* series from the Catalysts program reflects on the contribution of technological advances in cryo-EM to medically relevant studies.

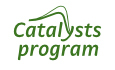

## Teaser

Structural biology is a field of research that investigates the structure and organization of macromolecules to decipher their functional mechanisms. Traditionally, it has adopted a reductionist approach out of necessity, focusing on the high-resolution analysis of macromolecules outside their natural cellular contexts. This contrasts sharply with clinical research, which examines human biology holistically through patient studies and cellular disease models, albeit with less detailed structural insights. Recent advancements are bridging this gap, enabling the study of macromolecular structures within more complex biological systems, from single cells to multicellular organisms and primary human tissues. These developments signal a pivotal shift within structural biology from traditional methodologies towards a more integrated understanding of biological complexity. Here, we highlight recent technological advancements that facilitate structural studies in tissue environments, showcasing the discoveries made possible with these approaches and the prospect of their applications in human clinical research.

Historically, structural biology has relied on techniques like X-ray crystallography, which, despite its precision, is limited by the need for crystallization, distancing studies from native biological conditions. The advent of cryo-electron microscopy (cryo-EM) in the late 20th century marked a significant advance, capturing macromolecules in their native state without the need for crystallization (Chua et al, 2022; Saibil, 2022). This has facilitated the structural analysis of larger and more complex macromolecular assemblies. However, most cryo-EM studies still adhere to a reductionist approach, focusing on macromolecules which are either recombinantly expressed or isolated from their native environments, due to the requirement of analyzing many 2D images of the same type of subject to derive high-resolution 3D structural information.

Cryo-electron tomography (cryo-ET) extends the capabilities of cryo-EM by allowing for the visualization of individual macromolecules in 3D (Förster and Briegel, 2024). This method collects a tilt-series of 2D cryo-EM images of a target at different angles, which are then used to calculate a 3D reconstruction of the imaged subject. Cryo-ET therefore offers a unique capability to conduct structural studies in complex cellular contexts, delivering crucial contextual information about spatial relationships, interactions within cells, and structures with low copy number (Turk and Baumeister, 2020; Hylton and Swulius, 2021; Hong et al, 2023; Theveny et al, 2022). Conventional 300 keV cryo-EM systems can only effectively image through samples thinner than about 500 nm—approximately the size of a typical bacterium. To facilitate exploration of thicker samples, cryo-focused ion beam (FIB) milling technology has been developed to thin down frozen samples, enabling structural studies within eukaryotic cells cultured in laboratory settings (Young and Villa, 2023).

Moreover, researchers have already set their sights beyond the individual cell and aim to investigate biological structures at a higher level of organization, within multicellular organisms or tissues. These larger samples offer the exciting possibility of connecting structural biology directly to human health and disease. However, larger samples also bring major challenges in sample preparation. The question of how to obtain high-resolution structural information within large, complex samples is currently a major focus of innovation for the future of bridging structural biology to clinical research.

**Figure 1:**
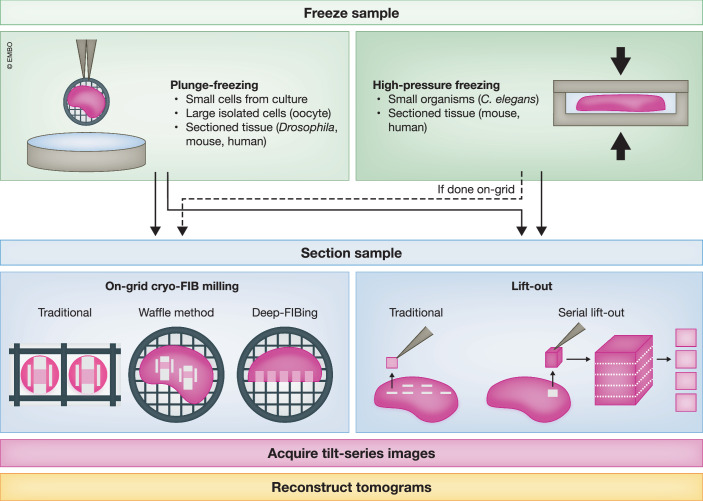
Sample preparation workflows enabling cryo-ET structural studies of frozen-hydrated multicellular samples. Upper: Sample freezing. High-pressure freezing is commonly employed to vitrify multicellular organisms and tissue samples. Plunge-freezing, typically used for vitrifying isolated cells on EM grids, can also be adapted with cryo-protectants to vitrify sectioned multicellular tissue samples on EM grids. Lower: Sample thinning. Cryo-FIB milling with different geometrical sequences and ion sources can produce lamellae from thick multicellular samples directly on the frozen grid. Alternatively, lift-out procedures enable the creation, and therefore imaging, of sample sections in different orientations compared to the on-grid lamellae. Moreover, the serial lift-out technique allows for the imaging of many adjacent sections of a multicellular sample.

The first breakthrough that has facilitated structural biology of large samples is in sample vitrification (Fig. 1, upper panel). The basic principle of cryogenic sample preparation is rapid freezing of samples to capture structures of interest in their native conformations within vitreous ice. For thin samples, this is routinely achieved through plunge-freezing (Dobro et al, 2010). However, thick samples prepared in this way freeze too slowly, leading to the formation of undesirable crystalline ice. The most widely used technique to vitrify thick samples is high-pressure freezing (HPF), which has allowed cryo-ET imaging of many large samples (Studer et al, 2008; Mahamid et al, 2015; Schaffer et al, 2019; Schiøtz et al, 2023; Birtasu et al, 2023; Gilbert et al, 2024; Zens et al, 2023; Klumpe et al, 2024; Dung et al, 2023; Matsui et al, 2024). However, compared to plunge-freezing, HPF is low-throughput and poses challenges for downstream FIB milling. Recently, a few groups have shown that it is possible to vitrify thick samples directly on EM grids through plunge-freezing with the addition of cryo-protectants (Bäuerlein et al, 2023; Creekmore et al, 2024; Jentoft et al, 2023). This approach significantly increases throughput and makes downstream FIB milling simpler, thereby making cryo-ET of thick samples more accessible.

The second breakthrough is thinning large samples for cryo-ET imaging (Fig. 1, lower panel). One established method for this purpose is the lift-out technique, where a small piece of the bulk sample is carved out by the FIB and physically lifted out for further thinning (Mahamid et al, 2015; Schaffer et al, 2019; Klumpe et al, 2024; Dung et al, 2023; Zens et al, 2023; Gilbert et al, 2024). Lift-out is commonly used for HPF samples but is technically difficult and time-consuming. A new approach, termed “serial lift-out”, increases throughput by allowing multiple thin sections to be produced from a single piece of lifted-out sample (in this case, an entire *Caenorhabditis elegans* worm) (Schiøtz et al, 2023). Another approach, dubbed the “waffle method”, is an alternative to lift-out and involves on-grid lamella milling of HPF samples (Kelley et al, 2022; Klykov et al, 2022; Birtasu et al, 2023; Matsui et al, 2024), providing another way to generate many lamellae from a bulk sample. Finally, two new approaches were developed to use FIB milling to first expose a vertical internal face within thick samples vitrified on-grid. This newly exposed face then enables lamella placement at various depths during subsequent FIB milling (Creekmore et al, 2024; Jentoft et al, 2023). This “deep-FIBing” technique is particularly useful for precisely targeting structures within different layers of thick samples as opposed to being restricted to surface-proximal targets. Regardless of the sample thinning approach chosen, the duration of FIB milling required to mill through thick samples is a major limitation. Gallium is the ion source most used for biological cryo-FIB milling, but it is relatively slow and becomes less precise at the high currents needed to mill large samples efficiently. In contrast, plasma-based ion sources (i.e., xenon plasma) mill faster and are more well-behaved at high currents, making them well-suited to large samples (Burnett et al, 2016; Smith et al, 2006). As plasma FIB instruments optimized for biological samples become more widely available (Kelley et al, 2023; Dumoux et al, 2023; Creekmore et al, 2024), plasma FIB milling will be an essential tool in the efficient generation of cryo-ET samples from tissue and other large samples.

The technological innovations outlined above have already led to several exciting proof-of-concept visualizations of biological structures within intact organisms or tissues. Lift-out techniques have provided insights into the organization of the multicellular worm *C. elegans* (Mahamid et al, 2015; Schaffer et al, 2019; Schiøtz et al, 2023). In addition, fruit fly nervous, epithelial, and reproductive tissues have been characterized by cryo-ET (Bäuerlein et al, 2023; Klumpe et al, 2024). In mice, multiple groups have visualized cardiomyocytes, brain synapses, and kidney glomeruli (Birtasu et al, 2023; Matsui et al, 2024). These efforts extend beyond model organisms into complex human cellular systems including forebrain organoids, oocytes, and the extracellular matrix of fibroblasts (Dung et al, 2023; Zens et al, 2023; Jentoft et al, 2023). Remarkably, primary human tissue has also been visualized by cryo-ET, revealing neurodegenerative disease protein aggregates within vitrified human brain tissue (Gilbert et al, 2024; Creekmore et al, 2024). It is even possible to use brain tissue vitrified directly at the time of autopsy (Creekmore et al, 2024), as opposed to using tissue flash-frozen in liquid nitrogen followed by storage at −80 °C (a common practice in tissue banking worldwide) which introduces crystalline ice and can damage fine cellular structures. These initial results are exciting, but they represent just the very tip of the iceberg of possibilities for in-tissue cryo-ET.

An important direction for the field will be further technological development at each step of the pipeline to make in-tissue cryo-ET more accessible and to increase throughput. At the level of sample acquisition, moving plunge freezers into hospital pathology laboratories or designing a new type of portable freezer would facilitate the study of human tissue samples collected through clinical biopsies to investigate the underlying mechanisms of disease. In addition, creating repositories of vitrified tissue similar to traditional tissue banks would expand access and options for clinically adjacent studies. For sample thinning, further development and automation of plasma FIB milling will greatly increase the throughput of sample preparation for cryo-ET. And finally, once data is acquired, a major challenge is conclusively identifying structures in the complex and crowded conditions within living cells and tissues. For this reason, most in situ studies to date have either focused on larger-scale ultrastructural characterization of tissues or have studied targets which are large, highly prevalent, symmetrical, and/or structurally distinct. One route for addressing this issue is to improve targeting techniques, including correlative light and electron microscopy (de Boer et al, 2015; Tian et al, 2021) as well as complementary approaches such as scanning electron microscopy during FIB milling (Wu et al, 2020), to consistently capture rare structures or events within FIB-milled lamellae. Another essential direction for future innovation is in computational techniques for identifying densities in crowded tomograms through advanced pattern mining, structure prediction, and template matching methods (Kim et al, 2023; Zeng et al, 2023; Cruz-León et al, 2024; Chen et al, 2023; Rice et al, 2023). It will also be critical to further develop infrastructure for storing and analyzing data on a larger scale.

There remain many significant challenges for doing structural biology in large and complex samples, and certainly the early results in these contexts still fall short of the atomic-level resolution achieved with the more standard, in vitro approaches. However, we are optimistic that with continued technology development and innovation, researchers will soon be able to obtain higher-resolution de novo structures from in-tissue cryo-ET studies, allowing for the examination of smaller and smaller structures in larger and larger contexts. This more holistic vision for structural biology will bring the field closer to the complexity of clinical research, unveiling structures and mechanisms pertinent to human health and disease in their native contexts that will augment our understanding of disease development and progression.
